# Bcl-2 Overexpression and Hypoxia Synergistically Enhance Angiogenic Properties of Dental Pulp Stem Cells

**DOI:** 10.3390/ijms21176159

**Published:** 2020-08-26

**Authors:** Waruna L. Dissanayaka, Yuanyuan Han, Lili Zhang, Ting Zou, Chengfei Zhang

**Affiliations:** 1 Applied Oral Sciences & Community Dental Care, Faculty of Dentistry, The University of Hong Kong, Hong Kong; u3006886@connect.hku.hk (Y.H.); liliz@connect.hku.hk (L.Z.); 2 Restorative Dental Sciences, Faculty of Dentistry, The University of Hong Kong, Hong Kong; zouting6@hku.hk

**Keywords:** Bcl-2, gene modification, angiogenesis, post-implantation cell survival, vascularization, tissue regeneration, dental pulp stem cells

## Abstract

Post-implantation cell survival and angio-/vasculogenesis are critical for the success of cell-based regenerative strategies. The current study aimed to overexpress B-cell lymphoma 2 (*Bcl-2*) gene in dental pulp stem cells (DPSCs) and examine the anti-apoptotic and angio-/vasculogenic effects both in-vitro and in-vivo. DPSCs were transduced with Bcl-2-green fluorescent protein (GFP) lentiviral particles and examined for cell proliferation and apoptosis. The cells were cultured under normoxic or hypoxic (0.5 mM CoCl_2_) conditions and examined for the expression of angiogenic factors and effects on endothelial cell proliferation, migration and vessel morphogenesis. Cells with or without hypoxic preconditioning were used in in-vivo Matrigel plug assay to study the post-implantation cell survival and angio-/vasculogenesis. Bcl-2-overexpressing-DPSCs showed significantly lower apoptosis than that of null-GFP-DPSCs under serum-free conditions. Under hypoxia, Bcl-2-overexpressing-DPSCs expressed significantly higher levels of vascular endothelial growth factor compared to that under normoxia and null-GFP-DPSCs. Consequently, Bcl-2-overexpressing-DPSCs significantly enhanced endothelial cell proliferation, migration and vascular tube formation on Matrigel. Immunohistological assessment of in-vivo transplanted Matrigel plugs showed significantly higher cell survival and vasculature in hypoxic preconditioned Bcl-2-overexpressing-DPSC group compared to null-GFP-DPSC group. In conclusion, Bcl-2 overexpression and hypoxic-preconditioning could be synergistically used to enhance post-implantation cell survival and angio-/vasculogenic properties of DPSCs.

## 1. Introduction

Dental pulp stem cells (DPSCs) are considered a promising population of stem cells in regenerative medicine for their ready availability and regenerative potential [1,2]. However, the survival and successful engraftment of engineered cellular/tissue constructs of DPSCs following implantation depends on adequate and timely supply of oxygen and nutrients and on the efficiency of removal of toxic waste [3]. Thus, the success of a tissue engineering strategy strictly relies on the rate of vascularization of the cellular/tissue construct mainly through angiogenesis.

It has been shown that capillaries can supply via diffusion only up to a distance of 200 µm and the cells located beyond this limit become hypoxic [4]. Cells have adapted to trigger an angiogenic response when they are faced with hypoxia or ischemia [5], which is mainly through secretion of vascular endothelial growth factor (VEGF) mediated by hypoxia inducible factors (HIFs) [6,7,8,9]. Under hypoxia, the degradation of HIF-1α subunit by prolyl hydroxylases (PHDs) is inhibited and consequently, VEGF expression is increased [10]. VEGF is one of the most potent angiogenic factors secreted in response to hypoxia [10,11,12]. It was shown that VEGF could induce the ingrowth of blood vessels into the ischemic tissues in both physiological and pathological conditions [9].

Despite this trigger, the spontaneous vascular ingrowth to tissue constructs following implantation is often limited from several tenths of micrometers to less than a millimeter per day [3]. Therefore, additional measures are required to enhance the cell viability until adequate blood supply is reached and to increase the rate of angiogenesis within an ischemic/hypoxic tissue construct.

In our previous study, we demonstrated that overexpression of anti-apoptotic gene B-cell lymphoma 2 gene (*Bcl-2*) is a viable approach to enhance cell viability of DPSCs [13]. *Bcl-2* is an anti-apoptotic gene, which was originally found to be overexpressed in human B-cell lymphoma [14]. It is localized at the outer membrane of mitochondria, where it plays a significant role in maintaining cell viability and inhibiting the actions of pro-apoptotic proteins. Subsequent studies demonstrated its effects on other aspects including cell differentiation, growth and angiogenesis [15,16,17,18,19,20,21]. Recombinant overexpression of the Bcl-2 protein has been demonstrated as a strategy to treat diseases associated with increased apoptosis such as cardiomyocyte death [15] and inherited cardiomyopathy [17,22]. Further, it has been shown that Bcl-2 overexpression enhances neuronal differentiation and could be used in the treatment of neurogenerative diseases [23,24,25].

In addition, several studies have shown that Bcl-2 overexpression enhances angiogenesis through HIF-1α mediated VEGF secretion in tumor cells [15,17,18,19,26]. Biroccio et al. has demonstrated that Bcl-2 and hypoxia can act synergistically to modulate VEGF expression and the in vivo angiogenic response in tumor cells [18]. Furthermore, it has been shown that upregulation of Bcl-2 in adipose derived stem cells enhances secretion of angiogenic growth factors including VEGF, fibroblast growth factor-2 (FGF-2) [27]. Recently, we demonstrated that hypoxic preconditioning is a viable approach to enhance angio-/vasculogenic properties of dental stem cells [28]. Nevertheless, it is unknown whether recombinant Bcl-2 overexpression could enhance the angiogenic potential of DPSCs.

Therefore, we hypothesized that overexpression of Bcl-2 in DPSCs could not only enhance the post-implantation cell survival but also the angiogenic properties through significant increase in VEGF secretion. Further, we postulated that hypoxic preconditioning would further enhance the angiogenic potential of Bcl-2 overexpressing DPSCs. Accordingly, in the current study we generated gene-modified DPSCs with Bcl-2 overexpression and examined their anti-apoptotic and angiogenic effects both under hypoxic and normoxic conditions in vitro and in vivo and demonstrated the potential mediatory role of HIF-1α transcription.

## 2. Results

### 2.1. Overexpression of Bcl-2 in Gene-Modified DPSCs

**Figure 1:**
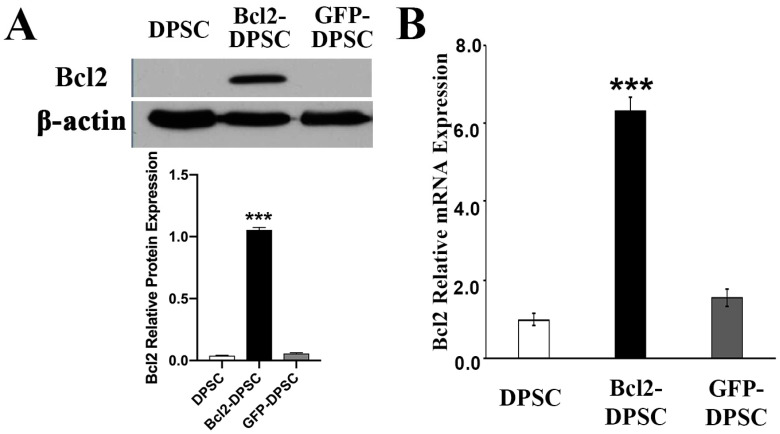
B-cell lymphoma 2 (*Bcl-2*) overexpression in dental pulp stem cells (DPSCs). (**A**) Western blot analysis and (**B**) real-time polymerase chain reaction of Bcl-2 protein and mRNA expression in Bcl-2- and green fluorescent protein (GFP)-transfected, and parental DPSCs. β-actin – endogenous control *** *p* < 0.001 versus the corresponding controls.

The transduction efficiency of gene-modified DPSCs was evaluated by calculating green fluorescent protein (GFP) positive cell percentage after 48-h lentiviral delivery. Over 85% DPSCs were found to be transduced as shown by the analysis (data not shown). Western blotting assay confirmed the overexpression of Bcl-2 in transduced DPSCs (Figure 1A) compared to that of null-GFP control and wild type groups, in which the expression was hardly detectable. The levels of mRNA expression further confirmed the *Bcl-2* overexpression in gene modified DPSCs (*p* < 0.05) (Figure 1B).

**Figure 2:**
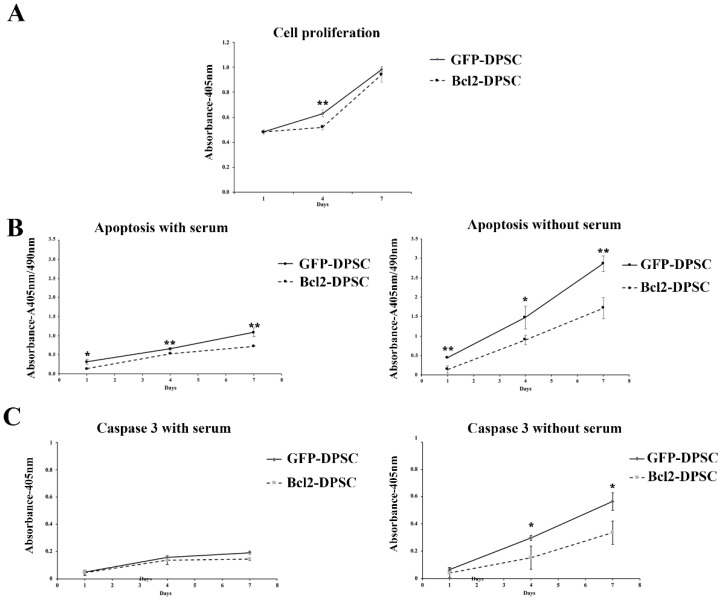
Characterization of Bcl-2-DPSCs. (**A**) Cell proliferation rates of Bcl-2-DPSCs and GFP-DPSCs as shown by Cell Counting Kit-8 (CCK-8) assay. Except at day 4, no significant difference was observed between the two groups. (**B**) Apoptosis in cultures of Bcl-2-DPSCs and GFP-DPSCs in the presence and absence of serum. Bcl-2-DPSCs demonstrated significantly lower apoptotic levels compared to GFP-DPSCs at all the time points under both conditions. Under serum starvation, the difference was markedly increased. (**C**) Caspase-3 activity in Bcl-2-DPSCs and GFP-DPSCs in the presence and absence of serum. Caspase-3 levels were significantly lower in Bcl-2-DPSCs in serum-free condition compared to that of GFP-DPSCs. * *p* < 0.05, ** *p* < 0.01versus the corresponding controls.

Further clarification of the gene modified DPSCs was carried out by assessing the anti-apoptotic nature of Bcl-2-overexpression. No significant difference in cell proliferation was observed in Bcl-2-DPSCs in comparison to that of GFP-DPSCs (Figure 2A) as shown by the Cell Counting Kit-8 (CCK-8) assay. Cell death assay (Figure 2B) and Caspase-3 assay (Figure 2C) showed significant anti-apoptotic activity in Bcl-2-DPSCs over GFP-DPSCs (*p* < 0.05). Apoptosis in both culture groups remained low when cultured with serum at all the time points. However, Bcl-2-DPSCs still showed a statistically significant (*p* < 0.05) lower level of apoptosis compared with that of GFP-DPSCs (Figure 2B). In the absence of serum, the difference in the levels of apoptosis between the two groups was markedly increased, as shown in both assays, with Bcl-2-DPSCs displaying significantly lower apoptotic rates than GFP-DPSCs (*p* < 0.05).

### 2.2. Bcl-2 Overexpression and Hypoxia Synergistically Increase VEGF Expression in DPSCs

**Figure 3:**
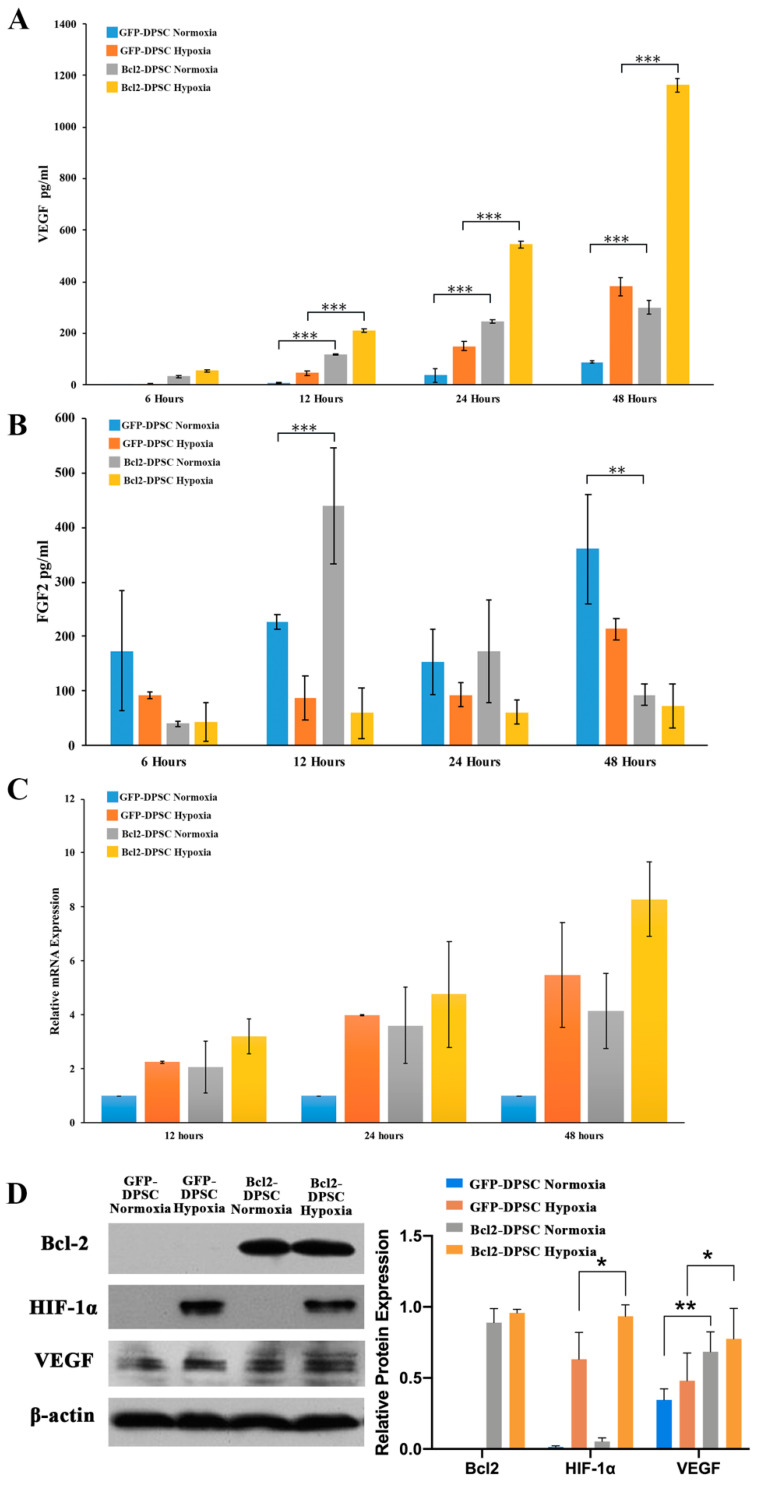
Expression of angiogenic factors in Bcl-2-DPSCs. Secretory (**A**) vascular endothelial growth factor (VEGF) and (**B**) fibroblast growth factor-2 (FGF2) levels at 6, 12, 24, and 48 h in Bcl-2-DPSCs and GFP-DPSCs under normoxia and hypoxia as detected by enzyme-linked immunosorbent assay (ELISA). (**C**) VEGF mRNA expression in Bcl-2-DPSCs and GFP-DPSCs under normoxia and hypoxia at 12, 24, and 48 h. (**D**) VEGF and hypoxia inducible factor (HIF)-1α protein levels at 24 h in Bcl-2-DPSCs and GFP-DPSCs under normoxia and hypoxia. β-actin – endogenous control * *p* < 0.05, ** *p* < 0.01, *** *p* < 0.001 versus the corresponding controls.

To examine whether the overexpression of Bcl-2 modulates the in vitro secretion of angiogenic factors, we measured the levels of VEGF and FGF2 proteins in the culture supernatants of the Bcl-2-DPSCs and GFP-DPSCs under normoxia and hypoxia. At 24 and 48 h, Bcl-2-DPSCs exhibited over threefold increase in VEGF levels compared to that of parental DPSCs in normoxia (*p* < 0.001) (Figure 3A). When hypoxia was introduced to cultures, the VEGF expression was significantly upregulated in both cell groups, while Bcl-2 transductants maintained its three-fold increase compared to the level of parental DPSCs (*p* < 0.001). In contrast, FGF2 protein levels showed a reduction under hypoxia in both GFP-DPSCs and Bcl-2-DPSCs (Figure 3B).

We further examined the mRNA and protein levels of VEGF in Bcl-2-DPSCs by qRT-PCR assays (Figure 3C) and western blotting (Figure 3D), respectively. The PCR results showed a similar pattern of mRNA expression to that of enzyme-linked immunosorbent assay (ELISA) results with highest levels of VEGF mRNA expression in Bcl-2 overexpressing DPSCs under hypoxia and lowest levels in GFP-DPSCs under normoxia. The total cellular VEGF protein levels examined by western blotting demonstrated the maximum VEGF expression at 24 h (Figure 3D). Moreover, HIF-1α expression was detected in both GFP-DPSCs and Bcl-2-DPSCs under hypoxia.

### 2.3. Bcl-2 Overexpressing DPSCs under Hypoxia Increase the Proliferation and Migration of Human Umbilical Vein Endothelial Cells (HUVECs)

**Figure 4:**
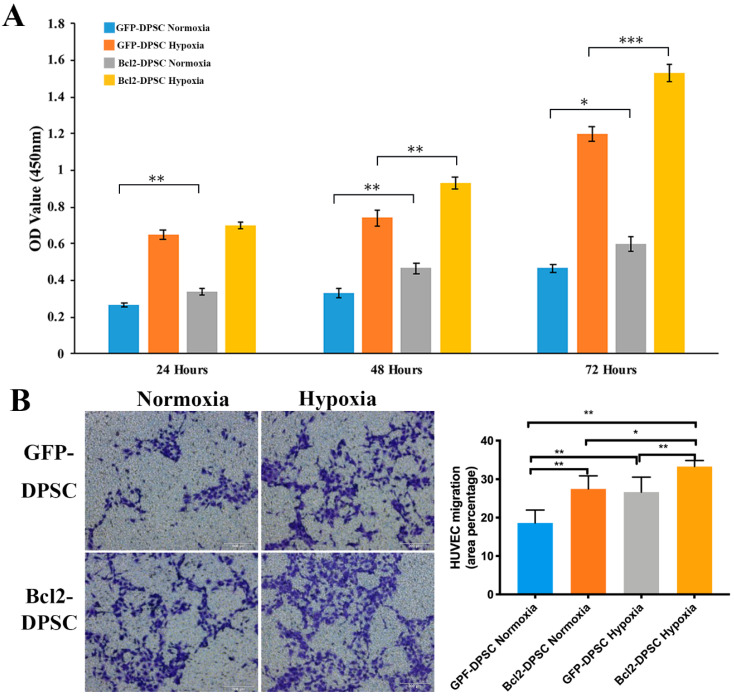
Paracrine effects of Bcl-2-DPSCs on human umbilical vein endothelial cell (HUVEC) proliferation and migration. (**A**) Cell proliferation rates as shown by CCK-8 assay and (**B**) Transwell migration assay of HUVECs cultured under conditioned media of Bcl-2-DPSCs and GFP-DPSCs collected under normoxia and hypoxia. * *p* < 0.05, ** *p* < 0.01, *** *p* < 0.001 versus the corresponding controls.

CCK-8 assay results (Figure 4A) showed that HUVECs cultured in conditioned media (CM) collected under hypoxia had a significantly higher (*p* < 0.05) proliferation than the cells cultured in the CM from normoxic conditions in both GFP-DPSC and Bcl-2-DPSC groups. Furthermore, cell proliferation of HUVECs in Bcl-2-DPSC CM under hypoxia was significantly higher than that of GFP-DPSC CM at 48 h (*p* < 0.01) and 72 h (*p* < 0.001). Interestingly, CM from Bcl-2-DPSCs in normoxia also resulted in significantly higher HUVEC proliferation rates (*p* < 0.05) at all the time points compared to that of GFP-DPSC CM in normoxia.

The results of transwell migration assay (Figure 4B) revealed that significantly higher number of HUVECs migrated to the bottom compartment in response to the Bcl-2-DPSC CM under hypoxia than CM from GFP-DPSCs both in normoxia (*p* < 0.01) and hypoxia (*p* < 0.05), and Bcl-2-DPSCs in normoxia (*p* < 0.01).

Taken together, the results confirmed that the paracrine signaling of Bcl-2 overexpressing DPSCs was significantly enhanced following hypoxic exposure.

### 2.4. Bcl-2 Overexpression and Hypoxia Synergistically Enhance Vascular-Like Tube Formation In Vitro

To examine whether the increase in VEGF secretion caused by Bcl-2 overexpression could enhance angiogenic properties of DPSCs, we performed Matrigel assay, which is considered a robust in vitro angiogenesis assay.

**Figure 5:**
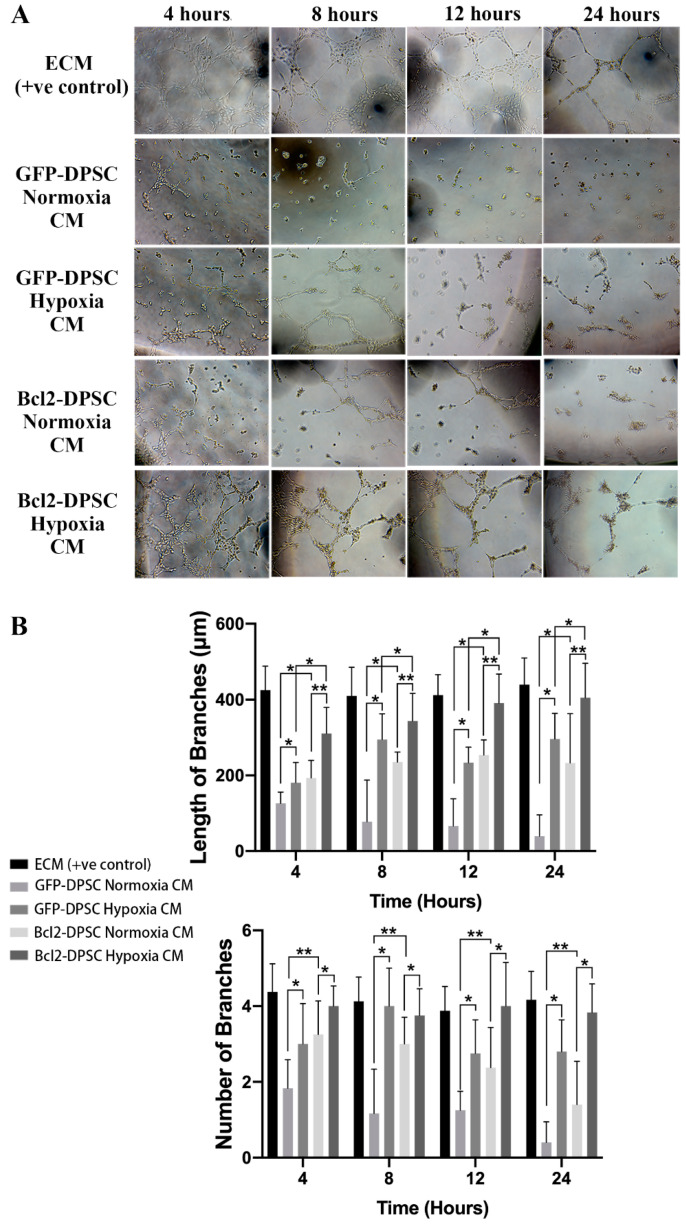
(**A**) Brightfield images of Matrigel tube formation assay. HUVECs cultured in conditioned media (CM) from Bcl-2-DPSCs under hypoxia formed a vascular network similar to that cultured in endothelial cell medium (ECM). (**B**) Quantification of length and number of vascular tubes formed by HUVECs. * *p* < 0.05, ** *p* < 0.01, versus the corresponding controls.

HUVECs seeded on Matrigel and cultured in CM from Bcl-2-DPSCs under hypoxia formed a network of capillary-like structures similar to that cultured in standard endothelial cell medium (ECM) (Figure 5A). In contrast, HUVECs cultured in CM from GFP-DPSCs both under normoxia and hypoxia, and Bcl-2-DPSCs under normoxia failed to form such tube network. CM from GFP-DPSCs did not support any tube formation, therefore, HUVECs remained as single cells. CM from GFP-DPSCs under hypoxia and Bcl-2-DPSCs under normoxia supported formation of few vascular structures. The statistical analysis (Figure 5B) showed that CM from Bcl-2-DPSCs under hypoxia group had a significantly higher number, as well as longer vascular tubes, compared to that of the other groups (*p* < 0.05). These findings resembled a similar pattern to the levels of VEGF secreted by different groups of DPSCs and, therefore, confirmed the functionality of enhanced VEGF levels caused by Bcl-2 overexpression.

### 2.5. Bcl-2 Overexpression and Hypoxic Preconditioning Synergistically Enhance Angiogenesis, Vasculogenesis, and Cell Viability In Vivo

**Figure 6:**
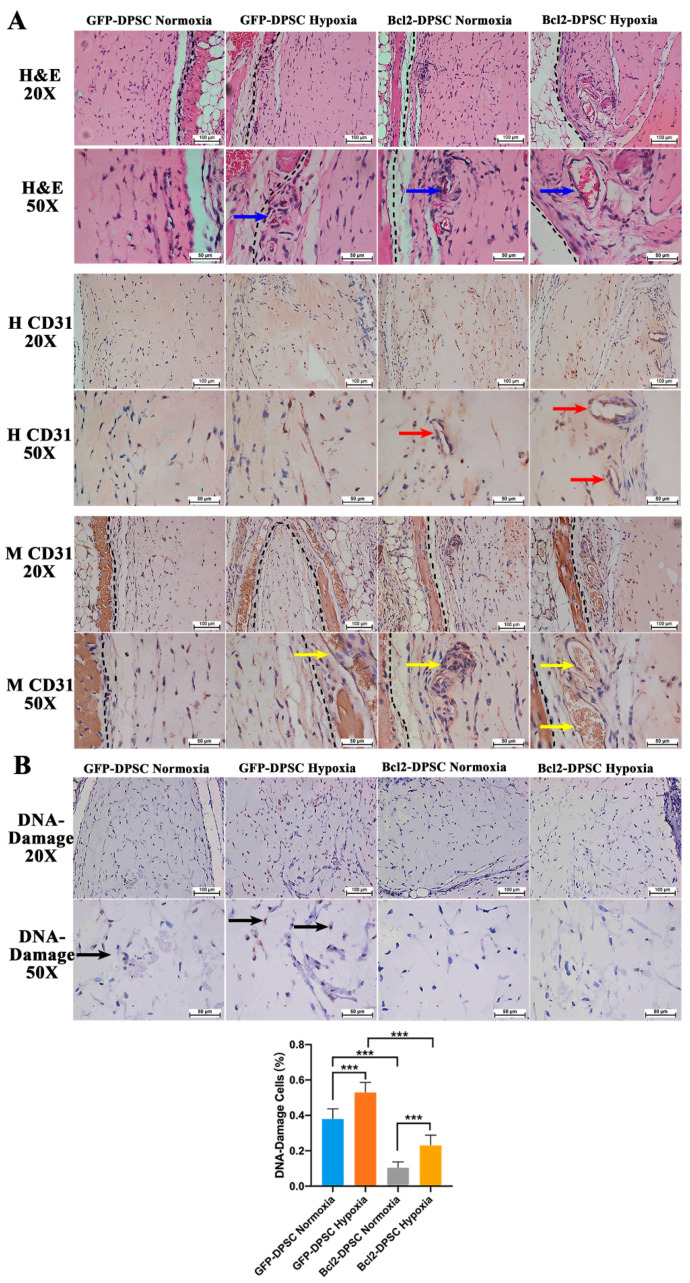
In vivo Matrigel plug assay: (**A**) Representative microscopic images of hematoxylin and eosin (H&E) (20× and 50× magnification) and immunohistochemistry for human CD31 (H CD31; 20× and 50× magnification) and mouse CD31 (M CD31; 20× and 50× magnification) of Matrigel plugs at 7 days of implantation. Broken black lines—interface between the mouse tissue and the Matrigel plug. Blue arrows—perfused blood vessels. Red arrows—human CD31 positive non-perfused lumens. Yellow arrows—mouse CD31 positive perfused blood vessels. (**B**) Representative microscopic images of immunohistochemistry for DNA damage (20× and 50× magnification) and quantified percentage cells with DNA damage. Black arrows—DNA damage positive cells. *** *p* < 0.001 versus the corresponding controls.

Histological evaluation of in vivo transplanted Matrigel plugs (Figure 6A) revealed increased vasculature with relatively larger blood vessels in hypoxic preconditioned Bcl-2-DPSC group compared to the small capillaries detected in normoxic Bcl-2-DPSCs and hypoxic preconditioned GFP-DPSC groups. Normoxic GFP-DPSC group did not show any recognizable perfused vessels within the plug. Immunohistochemistry for human CD31 (Figure 6A) detected positively stained endothelial-lined lumens both in hypoxic preconditioned and normoxic Bcl-2-DPSC groups while such lumens were not observed in any of the GFP-DPSC groups. Similarly, perfused blood vessels positive for mouse CD31 antibody (Figure 6A) were detected within both hypoxic preconditioned and normoxic Bcl-2-DPSC Matrigel plugs. In contrast, no mouse CD31 positive vessels were detected within hypoxic preconditioned or normoxic GFP-DPSC Matrigel plugs. These results represent the effects of respective VEGF levels secreted by the different groups. Hypoxic preconditioned Bcl-2-DPSCs, which showed the highest levels of VEGF secretion, demonstrated both vasculogenesis and angiogenesis in the Matrigel plugs. The non-perfused human CD31 positive lumens suggested endothelial differentiation of DPSCs and ongoing vasculogenic activity within Bcl-2-DPSC plugs. Simultaneously, mouse CD31 positive vessels indicated the host vascular ingrowth into the Matrigel plugs.

Immunohistochemistry for DNA damage antibody (Figure 6B) revealed significantly low number of cells with DNA damage in Bcl-2-DPSCs compared to GFP-DPSCs in Matrigel plugs, which confirmed the anti-apoptotic effects of Bcl-2 overexpression.

### 2.6. HIF-1α Is Partially Responsible for the Elevated VEGF Expression Levels in Bcl-2-DPSCs under Hypoxia

**Figure 7:**
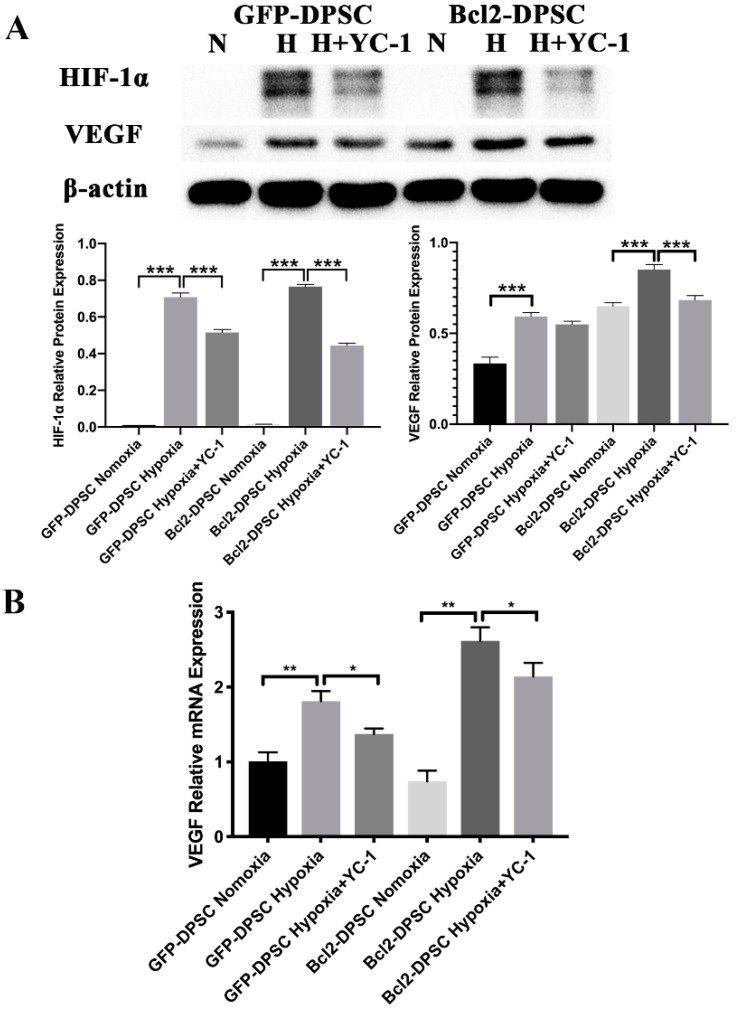
Role of HIF-1α in VEGF expression in Bcl-2-DPSCs under hypoxia. (**A**) Expression of protein levels of VEGF and HIF-1α in GFP-DPSCs and Bcl-2-DPSCs under normoxia (N), hypoxia (H), and YC-1 treatment as shown by western blotting. (**B**) Relative mRNA expression levels of VEGF in GFP-DPSCs and Bcl-2-DPSCs under normoxia (N), hypoxia (H), and YC-1 treatment as shown by qRT-PCR assay. β-actin – endogenous control * *p* < 0.05, ** *p* < 0.01 *** *p* < 0.001 versus the corresponding controls.

With the aim of elucidating the role of HIF-1α in Bcl-2 overexpression induced VEGF secretion, we used HIF-1α inhibitor YC-1 to inhibit expression of HIF-1α and examined the expression of VEGF in different groups of DPSCs. As shown in Figure 7A, YC-1 significantly inhibited HIF-1α expression in DPSCs under hypoxia. In turn, VEGF expression was also reduced as shown by mRNA (Figure 7B) and protein levels (Figure 7A) in both GFP-DPSCs and Bcl-2-DPSCs under hypoxia. These results indicated that the significant increase in VEGF in Bcl-2-DPSCs under hypoxia may be partially mediated by HIF-1α transcription.

## 3. Discussion

DPSCs are considered a promising cell source with a therapeutic potential in dental pulp regeneration and other tissue engineering approaches. Inadequate post-implantation cell survival and timely vascularization are two major barriers that need to overcome in utilizing these cells in regenerative strategies [3,21,28]. In the current study, for the first time, we demonstrated that Bcl-2 overexpression significantly enhances the post-implantation cell viability and VEGF mediated angiogenic and vasculogenic potential of DPSCs.

Many different factors, including necrosis, apoptosis, and lack of cytokine support, result in post-implantation cell death [29]. This directly affects the expected regenerative outcome as it largely depends on the number of active stem cells in the implant. We demonstrated that Bcl-2 overexpression could enhance the survival of DPSCs by reducing the apoptosis both under in vitro serum-free conditions and following in vivo implantation. Our findings are in accordance with previous studies that demonstrated Bcl-2 overexpression promotes the survival by decreasing apoptosis in the setting of hypoxia induced stress in tumors [18,30,31] or mesenchymal stem cells [21].

Once introduced to in vivo microenvironment, it is inevitable that the cells face low oxygen tension, therefore, in order to recapitulate the in vivo situation, we performed in vitro experiments under hypoxic conditions. We demonstrated a significant increase in VEGF expression under hypoxic conditions, particularly in Bcl-2-DPSCs. This finding also suggested the potential of hypoxic preconditioning as a mode of preparing stem/progenitor cells for in vivo implantation. We and others have previously reported that hypoxia preconditioning could enhance post-implantation cell survival [32] and angio-/vasculogenesis [28] by inducing HIF-1α.

Subsequently, we demonstrated the angiogenic effects of Bcl-2 overexpressing DPSCs under normoxic and hypoxic conditions, which was evident from the vascular structure formation in the in vitro Matrigel assay and functional angio-/vasculogenesis in transplanted Matrigel plugs in vivo. Our in vivo SCID mouse model of angiogenesis suggested that DPSCs overexpressing Bcl-2 were able to participate in inducing both angiogenic and vasculogenic response through at least 7 days. The Matrigel plugs of Bcl-2-DPSCs preconditioned to hypoxia showed the highest level of vasculature compared to the plugs containing GFP-DPSCs with or without hypoxic preconditioning. In addition, the number of cells with DNA damage in Matrigel plugs containing Bcl-2-DPSCs was significantly lower as compared with the controls. This suggests that overexpression of Bcl-2 in DPSCs has at least one of the following functions in vivo, neither of which is mutually exclusive. First, Bcl-2 protects DPSCs from apoptosis induced by physiological stress including hypoxia and ischemia following implantation in vivo. Second, Bcl-2 might potentiate the ability of DPSCs to differentiate into endothelial cells and functional blood vessels and also recruit host vessels into the plug through VEGF expression.

Although the role of Bcl-2 expression in the acquisition of angiogenic phenotype is not clear, it is logical to hypothesize that hypoxia acts as a common signal to prolong the expression of angiogenic genes during Bcl-2 overexpression. Therefore, as tissue hypoxia is known to prime cells to express VEGF, Bcl-2 overexpression could provide required signal to enhance or maintain this state of angiogenic factor production. We examined the expression levels of FGF-2 and VEGF in Bcl-2-DPSCs as these are two of the most significant angiogenic growth factors, which were reported to be associated with Bcl-2 overexpression [33,34]. In contrast, our results showed an upregulation only in VEGF expression under hypoxia.

Our results were in accordance with the evidence that the HIF-1α production is correlated with the production of VEGF. Several studies have proposed distinct molecular mechanisms that regulate VEGF mediated angiogenic effects of Bcl-2 overexpression [10,12,17,18,19]. One study has shown that Bcl-2 overexpression could stabilize VEGF mRNA and HIF-1α mediated VEGF transcription under hypoxia [19]. Furthermore, it has been shown that Bcl-2 overexpression inhibits mitochondrial metabolism and HIF-1α hydroxylase, which cleaves HIF-1α under high oxygen tension and is inactive in hypoxia [35]. Thus, Bcl-2 has been shown to activate HIF-1α expression by removing HIF-1α hydroxylase. Additionally, a previous study has demonstrated that Bcl-2 overexpressing melanoma cells, under low oxygen conditions, induce the HIF-1α cascades through modulation of nuclear factor κB (NF-κB) and other transcription factors to induce transcriptional activation of VEGF [36,37]. Therefore, it is possible that, Bcl-2 protein can directly and indirectly modify post-translational hydroxylation of HIF-1α and induce angiogenesis [38].

Our results showed that HIF-1α transcription could only be partially responsible for the increased VEGF expression in Bcl-2-DPSCs under hypoxia. Moreover, we did not observe any expression of HIF-1α in Bcl-2-DPSCs under normoxia, despite the increased VEGF expression. Both these observations are suggestive of Bcl-2 could increase VEGF expression independent of HIF-1α transcription, as well. In supportive of our observations, it has been shown that Bcl-2 expression also induces angiogenesis through down-regulation of p53 [39] or VEGF mRNA stabilization [19] in tumor cells. Further analysis, therefore, is required to evaluate whether Bcl-2 mediates the pro-angiogenic effects in DPSCs through other hypoxic regulatory factors of VEGF promoter, by stabilization of VEGF mRNA or through a totally different pathway.

Although Bcl-2 is considered an anti-apoptotic gene and its overexpression is commonly associated with different types of human cancers [17,18], there is no solid evidence to show that Bcl-2 overexpression alone will induce carcinogenesis. In relation to several cancers, it has been shown that Bcl-2 overexpression alone does not cause cancer, but simultaneous overexpression of an oncogene is required to induce carcinogenesis [40,41]. In fact, a study has shown that expression of Bcl-2 during liver carcinogenesis results in a delay of progression rather than an increase in tumorigenesis [41]. It has also been suggested that although Bcl-2 is anti-apoptotic, Bcl-2 overexpression does not completely prevent apoptosis in the cells [25].

Despite our findings that Bcl-2 overexpressing DPSCs under hypoxia could enhance angio-/vasculogenesis, some challenges remain to be addressed further. First, the specific mechanisms behind Bcl-2 overexpression mediated anti-apoptotic and angiogenic effects in the transplanted cells need to be elucidated. Second, observation of long-term effects of Bcl-2 overexpression in DPSCs is necessary before considering the use of this approach in translational applications. Third, the signaling pathways involved in the Bcl-2 mediated VEGF expression must be further clarified. Finally, non-viral methods need to be developed in order to facilitate the safe and effective gene modification to achieve Bcl-2 expression in DPSCs.

## 4. Materials and Methods

### 4.1. Isolation and Culture of Human DPSCs

The experiments that used cells isolated from human patients were approved by the institutional review board of the University of Hong Kong. After obtaining the informed consent, DPSCs were isolated from the freshly extracted third molars of human dental patients aged between 18 and 25 years. Briefly, after cleaning the surfaces, the teeth were cut at the cementoenamel junction using a sterile fissure bur on a hand-piece to reveal the pulp chamber. The pulp tissue was gently separated from the crown and root, cut into small pieces and digested in a solution containing 3 mg/mL collagenase type I (GIBCO-Invitrogen, Carlsbad, CA, USA) and 4 mg/mL dispase (GIBCO-Invitrogen) for 1 h at 37 °C. The digested solution was passed through a 70-μm strainer (BD Biosciences, Franklin Lakes, NJ, USA) to obtain single-cell suspensions. After washing with phosphate buffered saline (PBS), the cells were seeded in 75-cm^2^ culture flasks containing α-minimum essential medium (α-MEM) supplemented with 15% fetal bovine serum, L-ascorbic acid-2-phosphate, and 100 U/mL Penicillin-G, 100 mg/mL streptomycin and cultured under 5% CO_2_ at 37 °C. The isolated cells were characterized for their “stemness” by flow cytometry analysis of the expression of typical mesenchymal stem cell markers (positive for CD73, CD90, CD105, STRO-1, and negative for CD45), as well as by multilineage differentiation assays with osteo-/odontogenic, adipogenic, and neurogenic induction media. The relevant results have been reported in one of our previous studies [2]. DPSCs isolated from one human donor were used in subsequent triplicate experiments. Human umbilical vein endothelial cells (HUVECs) were purchased commercially (ScienCell Research Laboratories, San Diego, CA, USA) and cultured in endothelial cell medium (ECM; ScienCell Research Laboratories) at 37 °C with 5% CO_2_. Cells from passages 3 to 6 of each cell type were used in all experiments according to the parameters described below. Hypoxic condition in cell cultures was induced by addition of 0.5 mM CoCl_2_ (Sigma-Aldrich Corporation, St. Louis, MO, USA) into the medium.

### 4.2. Overexpression of Bcl-2 in DPSCs

Premade human target, Bcl-2-GFP lentiviral particles and respective null-GFP-control particles were commercially purchased from GenTarget Inc, San Diego, CA, USA. DPSC transfection with *Bcl-2* was carried out according to the manufacturer’s protocol. Briefly, DPSCs of passage 3 were seeded at 0.5 × 10^5^/mL 0.5 mL in a 24-well plate and incubated overnight. At 70% confluence, the culture medium was replaced with 0.5 mL fresh complete medium, and then 50 µL of 1 × 10^8^ infectious units (IFU)/mL lentivirus stock was added. After incubating the cultures for 72 h in a 37 °C/CO_2_ incubator, transfection rate was checked under fluorescence microscope. Stable Bcl-2 overexpressing cells were selected by adding puromycin. The required puromycin concentration was determined from the antibiotic’s killing curve for DPSCs as obtained through a pilot experiment. For simplicity, the Bcl-2-GFP overexpressing cells and null-GFP-control cells will be referred to as Bcl-2-DPSCs and GFP-DPSCs, respectively. Puromycin selected transgenic cells were examined for *Bcl-2* expression by quantitative real-time polymerase chain reaction (qRT-PCR) and western blot assays as described below.

### 4.3. Cell Proliferation Assays

#### 4.3.1. Bcl-2-DPSCs and GFP-DPSCs

GFP-DPSCs and Bcl-2-DPSCs were inoculated in a 96-well plate at a concentration of 1 × 10^4^ cells per well and incubated in a 37 °C, 5% CO_2_ humidified incubator. Ten microliters of the CCK-8 solution (Sigma-Aldrich) was added to each well of the plate. After incubating the plate for 4 h in the incubator, the absorbance at 450 nm was measured using a microplate reader.

#### 4.3.2. Endothelial Cell Proliferation

Proliferation rates of HUVECs in different CM collected from GFP-DPSCs and Bcl-2-DPSCs under normoxia and hypoxia were assessed by CCK8 assay as described above.

### 4.4. Apoptosis Assays

#### 4.4.1. Cell Death ELISA Assay

GFP-DPSCs and Bcl-2-DPSCs were seeded on a 48-well plate at a density of 1 × 10^4^ cells per well. At 80–90% confluence, the medium was changed to serum-free medium and the cells were starved for 12 h. At various time intervals, cell lysates were harvested for consequent analyses. Cell Death Detection ELISA Plus Kit (Roche Diagnostics GmbH, Mannheim, Germany) was used to measure the cell apoptosis. Based on the manufacturer’s protocol, 200 μL of lysis buffer was added to each well and incubated for 30 min at room temperature. The lysate was centrifuged at 200× *g* for 10 min and 20 μL from the supernatant was transferred to the streptavidin coated microplate wells. After adding 80 μL of the immunoreagent (mixture of Anti-histone-biotin and Anti-DNA-POD), the microplate was covered with an adhesive cover foil and incubated on a microplate shaker under gentle shaking at room temperature for 2 h. Each well was rinsed three times with 300 μL incubation buffer and solutions were removed thoroughly by tapping. After pipetting 100 μL of 2,2′-Azinobis (3-ethylbenzothiazoline-6-sulfonic acid) diammonium salt (ABTS) solution to each well, the microplate was incubated on a plate shaker at 250 rpm for 20 min. The reaction was stopped by adding 100 μL of ABTS stop solution and the absorbance at 405 nm was measured using a microplate reader. The well with only ABTS and ABTS stop solution was measured as the blank.

#### 4.4.2. Caspase-3 Assay

After culturing the cells as described above under 4.4.1, Caspase-3 activity of the GFP-DPSCs and Bcl-2-DPSCs was measured using Caspase-3 Colorimetric Assay kit (R&D Systems, Inc., Minneapolis, MN, USA). Briefly, the Lysis Buffer was added to collect the whole-cell lysate. Then, the cell lysate was incubated on ice for 10 min, centrifuged and the supernate was transferred to a new tube. After adding 50 μL of cell lysate, 50 μL of 2× Reaction Buffer, and 5 μL of Caspase-3 colorimetric substrate to each well, the microplate was incubated for 2 h at 37 °C. Using a microplate reader, readings were taken at wavelength of 405 nm. No cell lysate and no substrate groups were used as controls of the assay.

### 4.5. Expression of Angiogenic Proteins

#### 4.5.1. ELISA Assays

The amount of VEGF and FGF2 proteins in the supernatant (at 6, 12, 24 and 48 h of hypoxia) of GFP-DPSCs and Bcl-2-DPSCs cultured under normoxia and hypoxia was determined with ELISA kits (R & D Systems, Minneapolis, MN, USA) according to the manufacturer’s instructions. Firstly, the 96-well microplate was coated overnight with 100 μL capture antibody (diluted in PBS) and blocked for 1 h with 300 μL Reagent Diluent (1% bovine serum albumin (BSA) in PBS). After washing 3 times with Wash Buffer (0.05% Tween 20 in PBS), 100 μL of samples or standards diluted in Reagent Diluent per well were added to the microplate and incubated for 2 h at room temperature. After repeating the washing step, 100 μL Detection Antibody (diluted in Regent Diluent) was added to each well and incubated for 2 h at room temperature. Then, 100 μL of the working dilution of Streptavidin- horseradish peroxidase (HRP) was added and incubated for 20 min avoiding direct light. Subsequently, 100 μL Substrate Solution was added, incubated for 20 min, 50 μL Stop Solution was added to each well and the plate was tapped gently for mixing. Finally, the optical density of each well was determined immediately under 450 nm and 540 nm by SpectraMax^®^ M2 microplate reader (Molecular Devices LLC, San Jose, CA, USA). The readings at 540 nm were subtracted from the readings at 450 nm and the concentrations of unknown samples were calculated using the standard curve. The results were expressed as pg protein/mg total protein.

#### 4.5.2. VEGF mRNA and Total Protein Expression

Cells were seeded at a density of 3 × 10^5^ cells/mL in six-well plates and cultured for 3 days to achieve 80–90% confluence. Then, the cells were starved with serum-free medium for 12 h, followed by incubation with 0.5 mM CoCl_2_ (Sigma-Aldrich). Total mRNA and cellular protein were harvested after 24 h for subsequent qPCR and western blot assays, respectively, as described below.

### 4.6. Conditioned Media (CM)

GFP-DPSCs or Bcl-2-DPSCs were cultured on 75-cm^2^ flasks until 80% confluence in standard culture medium and changed to serum-free medium. Then, the cells were starved with serum-free medium for 12 h, followed by incubation with 0.5 mM CoCl_2_ (Sigma-Aldrich). Conditioned media under normoxia and induced hypoxia were collected after 24 h, centrifuged at 1500 rpm, and filtered to remove cell debris. Despite the fact that maximum secretory VEGF expression was observed at 48 h in Bcl-2-DPSCs under hypoxia, conditioned medium was collected at 24 h in order to avoid the medium getting severely depleted of vital components required for cell growth.

### 4.7. Transwell Migration Assay

Twenty-four well transwell permeable supports with 8 μm pores (Thermo Scientific, Rockford, IL, USA) were used. HUVECs were trypsinized, resuspended by serum-free medium, and seeded on the upper chamber of inserts at a density of 1.5 × 10^5^/150 µL. Then, 500 µL of CM collected from GFP-DPSCs or Bcl-2-DPSCs under normoxia or hypoxia was added to the lower compartment of each well. After 24 h, the medium was removed and the upper side of the permeable membrane was cleansed with a cotton swab to remove the cells that have not passed through the membrane. The cells on the lower surface of the membrane were fixed by 4% paraformaldehyde for 30 min and stained with 0.1% (*w/v*) crystal violet (Sigma-Aldrich) for 15 min. The number of cells on the lower surface of the permeable membrane was calculated under the inverted microscope (Nikon, Tokyo, Japan) and average of three fields per each well was taken as the final value.

### 4.8. Vascular Morphogenesis Assay on Matrigel

Unpolymerized Matrigel (10 mg/mL, Becton Dickinson, Milan, Italy) was placed in a 24-well microtiter plate (300 µL/well) and allowed to polymerize for 1 h at 37 °C. HUVECs were plated at 2 × 10^5^ cells/well in 1 mL of ECM (positive control) or CM collected from GFP-DPSCs or Bcl-2-DPSCs under normoxia or hypoxia. After incubation in a 5% CO_2_ humidified atmosphere at 37 °C, formation of tubes was observed through an inverted, phase-contrast light microscope at hourly intervals for 24 h. Images were captured at 4, 8, 12, and 24 h after cell seeding and vascular tube length and number were calculated with NIS-Elements AR 3.1 software (Nikon).

### 4.9. In Vivo Matrigel Assay

The animal experimental procedures were approved by the Committee on the Use of Live Animals in Teaching and Research (CULATR) of the University of Hong Kong. All animal experiments were performed in accordance to the Guide for the Care and Use of Laboratory Animals published by the U.S. National Institutes of Health and regulations at the Laboratory Animal Unit of the University of Hong Kong. To evaluate the ability of Bcl-2 to modulate the neovascularization, in vivo Matrigel plug assay was performed. Briefly, Bcl-2-DPSCs and GFP-DPSCs either cultured under normal conditions or in medium supplemented with 0.5 mM CoCl_2_ for 24 h were encapsulated in growth factor reduced Matrigel (Becton Dickinson, Bedford, MA, USA). A volume of 600 μL cell-Matrigel suspension was injected subcutaneously into the flank of 8-wk-old severe combined immunodeficient (SCID) mice bilaterally, where it rapidly formed a gel. After 7 days of injection, mice were euthanized, Matrigel plugs were retrieved and angiogenic response was evaluated by histological and immunohistochemical analysis.

#### Histology and Immunohistochemistry

The whole Matrigel plug was sectioned vertically (from the skin to muscle direction) and 5 sections were stained from each plug. Hematoxylin and eosin staining was performed to examine the morphology and structure of the vascular tissues generated in Matrigel plugs. Immunohistochemistry (IHC) was performed using Mouse and Rabbit Specific HRP/ 3,3′-Diaminobenzidine (DAB) (ABC) Detection IHC kit (Abcam, Cambridge, UK). Vasculature was examined on histological sections stained with rabbit anti-human CD31 (Abcam, 1:500); rabbit anti-mouse CD31 (Abcam, 1:500); recombinant rabbit IgG, monoclonal - isotype control (Abcam, 1:500). Accordingly, DNA damaged cells were detected with the staining for mouse anti-DNA/RNA damage antibody (Abcam, 1:500). Goat anti-polyvalent antibody (from the Abcam IHC kit) was used as the secondary antibody. The microscopic imaging (Nikon Eclipse LV100N POL) was performed to capture the expression patterns. DNA damaged cell percentage (DNA-damage cell numbers/total cell numbers) was determined by randomly selecting 8 areas in each section and calculating the numbers of positively stained cells and total cells. Human and mouse CD31 positive lumens were identified using NIS-Elements AR 3.1 software (Nikon, Tokyo, Japan). Any brown-stained endothelial cell or endothelial cell cluster, clearly separates from adjacent microvessels and cells, were considered a single, countable microvessel. Further, the identified perfused vessels were confirmed by comparing to the vessels in the mouse tissue that contained red blood cells under physiological conditions.

### 4.10. Inhibition of HIF-1α

To assess the role of HIF-1α in expression of VEGF in Bcl-2-DPSCs under hypoxia, cells were cultured in 6 cm culture dishes until 80% confluence and transferred to a hypoxia incubator flushed with 1% O_2_, 5% CO_2_, and 94% N2 and incubated for 24 h. For inhibition experiments, the cells were pretreated with 80 mM YC-1 (Sigma-Aldrich) for 2 h, followed by hypoxic stimulation. Total RNA and proteins were extracted for subsequent qRT-PCR and western blot assays, respectively.

### 4.11. Quantitative Real-Time PCR Assay

Total cellular RNA was isolated using a RNeasy Mini kit (Qiagen, Crawley, UK), and was quantified by a spectrophotometer. Complementary DNA synthesis was carried out by SuperScript III Reverse transcriptase (Invitrogen). DNA amplification was performed using ABI Prism 7000 sequence detection system (Applied Biosystems) with SYBR green (Applied Biosystems). The primer sequences (Sigma-Aldrich) used are as follows: *Bcl-2* (NM_000633) F 5′-GAGACAGCCAGGAGAAATCA-3′, R 5′-CCTGTGGATGACTGAGTACC-3′, *HIF-1α* (NM_001530) F 5′-GGCGCGAACGACAAGAAAAAG-3′, R 5′-CCTTATCAAGATGCGAACTCACA-3′, *VEGF* (NM_001171623.1)—F 5′-CAAAAACGAAAGCGCAAGAAA-3′, R 5′-GCGGGCACCAACGTACAC-3′, *GAPDH* (NM_001256799.3)—F 5′-GGCATGGACTGTGGTCATGAG-3′, R 5′-TGCACCACCAACTGCTTAGC-3′. Expression of Glyceraldehyde 3-phosphate dehydrogenase (GAPDH) was used as an internal standard for RNA loading.

### 4.12. Western Blotting

Cell lysates were extracted by adding M-PER Mammalian Protein Extraction Reagent (Thermo Scientific) to the cell cultures and incubating them on ice for 20 min. After calculating the protein concentrations using a BCA protein assay kit (Thermo Scientific), the protein samples (50 µg) were subjected to 12% SDS-PAGE (Bio-Rad Laboratories) and transferred to nitrocellulose membranes (Bio-Rad, Melville, NY, USA). Nitrocellulose membranes were blocked in PBS containing 0.05% Tween 20 and 3% nonfat dry milk and then incubated with a mouse monoclonal anti-HIF-1α antibody (BD Biosciences) diluted 1: 500, anti-Bcl-2 (Santa Cruz Biotechnology, Inc., Santa Cruz, CA, USA) diluted 1: 1000, anti-VEGF (Abcam) diluted 1: 1000, anti-β-actin antibodies (Santa Cruz Biotechnology) overnight at 4 °C. Following three 15-min washes in TBS-T (Tris-buffered saline, 0.1% Tween 20) with 0.05% Tween 20, blots were incubated with horseradish–peroxidase-conjugated rabbit anti-mouse antibody at a 1:10,000 dilution for 2 h. The membranes were developed using enhanced chemiluminescence reagents (Thermo Scientific), and the density of bands was analyzed by Quantity One 4.6.9 software (Bio-Rad Laboratories). The protein expression levels were normalized to that of β-actin (Santa Cruz Biotechnology, Inc.) levels.

### 4.13. Statistical Analysis

All the results are presented as mean ± standard deviation (SD). Statistical analysis was carried out using Student’s *t*-test for assays done between two groups and one-way ANOVA with a Tukey’s post-hoc test in multiple comparisons. Statistical significance was considered at *p* < 0.05.

## 5. Conclusions

In the current study, we demonstrated that DPSCs overexpressing Bcl-2 significantly enhanced the VEGF secretion when exposed to hypoxic condition and reduced the post-implantation cell death. Hypoxic or ischemic environment under normal circumstances imposes severe constraints on endothelial cell growth and survival. Our findings suggest an approach whereby Bcl-2 induced expression of VEGF under hypoxic preconditioning may function to enhance the angio-/vasculogenesis in the oxygen-deficient in vivo microenvironment that would facilitate the uninterrupted flow of nutrients to the implant. However, further studies are necessary to determine the specific mechanisms of Bcl-2 overexpression mediated effects in the transplanted cells. Gene modification to achieve Bcl-2 expression using a non-viral vector could be an effective and safe approach for obtaining desired effects in terms of post-implantation cell survival and vascularization.
